# Three new cecidogenous species of *Palaeomystella* Fletcher (Lepidoptera, Momphidae) from the Brazilian Atlantic Rain Forest

**DOI:** 10.3897/zookeys.433.7379

**Published:** 2014-08-13

**Authors:** Fernando A. Luz, Gislene L. Gonçalves, Gilson R. P. Moreira, Vitor O. Becker

**Affiliations:** 1 PPG Ecologia, Departamento de Ecologia, Instituto de Biociências, Universidade Federal do Rio Grande do Sul, Av. Bento Gonçalves 9500, Porto Alegre, RS, 91501-970, Brazil; 2 PPG Biologia Animal, Departamento de Zoologia, Instituto de Biociências, Universidade Federal do Rio Grande do Sul, Av. Bento Gonçalves, 9500, Porto Alegre, RS 91501-970, Brazil; 3 Instituto de Alta Investigación, Universidad de Tarapacá, Antofagasta 1520, Arica, Chile; 4 Departamento de Zoologia, Instituto de Biociências, Universidade Federal do Rio Grande do Sul, Av. Bento Gonçalves 9500, Porto Alegre, RS, 91501-970, Brazil; 5 Reserva Serra Bonita, P.O. Box 001, Camacan, BA 45880-970, Brazil

**Keywords:** Melastomataceae, melastome, *Mompha*, momphines, momphid moths, Neotropical region, plant galls, *Tibouchina*

## Abstract

Three new cecidogenous species of *Palaeomystella* Fletcher (Lepidoptera, Momphidae) from the Brazilian Atlantic Rain Forest are described. Larvae of *P. fernandesi* Moreira & Becker, **sp. n.**, *P. rosaemariae* Moreira & Becker, **sp. n.** and *P. tavaresi* Becker & Moreira, **sp. n.** induce galls, respectively, on *Tibouchina sellowiana* (Cham.) Cogn., *T. asperior* (Cham.) Cogn. and *T. fissinervia* (Schrank & Mart. ex DC.) Cogn. (Melastomataceae). Adults, immature stages and galls are illustrated, and data on life history and a preliminary analysis of mitochondrial DNA sequences, including related species, are also provided.

## Introduction

Cecidogeny has evolved independently in at least 20 microlepidopteran families, mostly within the Gelechioidea. Although they total only a few hundred species, the majority of these moths have been associated with their gall morphotype only, as most are still awaiting taxonomic description at the specific level (Miller 2005). The Neotropical Melastomataceae host a variety of morphotype galls that are known to be induced primarily by Lepidoptera. In this case, even the family identity of the corresponding gall-inducers remained uncertain for a long time (e.g., Tavares 1917, Houard 1933, Lima 1945) and was only recently associated with the momphine lineage as a subfamily of Coleophoridae (Becker 1999) and is herein treated as Momphidae (*sensu* van Nieukerken et al. 2011, Heikkilä et al. 2013). Only four of these species have been described, all belonging to *Palaeomystella* Fletcher, 1940 (Becker 1999, Becker and Adamski 2008). The type-species (*Palaeomystella chalcopeda* Meyrick, 1931), for which only the female holotype from Nova Friburgo, Brazil is known, has not yet been associated with any gall morphotype (Becker 1999). The other three species such as *Palaeomystella tibouchinae* Becker & Adamski, 2008 and *Palaeomystella oligophaga* Becker & Adamski, 2008 induce galls on species of *Tibouchina* Aubl. and *Macairea* DC. in the Cerrado biome in central Brazil, and *Palaeomystella henriettiphila* Becker & Adamski, 2008 induces galls on a species of *Henriettea* DC. in northeast Brazil (Becker and Adamski 2008). Other similar gall morphotypes occurring in Brazil have been reported for Melastomataceae as induced by unidentified Lepidoptera larvae (e.g., Gonçalves-Alvim et al. 1999, Maia and Fernandes 2004, Carneiro et al. 2009, Santos et al. 2011, Bena and Vanin 2013, Ferreira and Isaias 2013, Isaias et al. 2013, Vecchi et al. 2013). Thus, these aspects increase the urgency of alpha-taxonomic work with this specialized moth lineage, together with descriptions of the gall morphotypes that they induce.

The majority of the gall morphotypes (ca. 30) that are known to be induced by unidentified lepidopteran larvae in Brazil were originally described from the Atlantic Rain Forest (Rio de Janeiro State), mostly on species of *Tibouchina* (Tavares 1917, Houard 1933). In fact, this is one of the most diverse genera within the Melastomataceae that occur in this biome, totaling at least 137 species in southern Brazil, where most are endemic (Goldenberg et al. 2012, Guimarães 2014). Although now substantially reduced and fragmented, the Atlantic Rain Forest still supports one of the most diverse communities of plants and animals on earth, with high endemism (for general descriptions and discussions, see Morellato and Haddad 2000, Myers et al. 2000, Carnaval et al. 2009). Thus, as pointed out for this biome by Brito et al. (2013), regarding the expected diversity of leaf miner moths in general, it is expected that several species of cecidogenous momphine moths associated with Melastomataceae await description.

In the course of an ongoing survey on the diversity of microlepidopterans in the Atlantic Rain Forest, Brazil, three momphid species associated with galls induced on three different species of *Tibouchina* were found recently: one morphotype in Bahia and two others in Rio Grande do Sul. A comparison between their inducers and type material not only revealed the generic affinity of these microlepidopterans with *Palaeomystella*, but also indicated that they have diagnosable, stable, distinctive characters. Therefore, three new species are proposed here; their last larval instar, pupal and adult stages are described and illustrated, and their life history, including a general description of their galls, is characterized. A preliminary phylogenetic inference based on mitochondrial DNA sequences, including additional members of the genus, is also presented.

## Materials and methods

Adults used in the study were reared by the first three authors from galls maintained in small plastic vials under controlled abiotic conditions (14 h light / 10 h dark; 25 ± 2 °C) in the Laboratório de Morfologia e Comportamento de Insetos, Departamento de Zoologia, Universidade Federal do Rio Grande do Sul (UFRGS), Porto Alegre city, Rio Grande do Sul State (RS), Brazil, from March 2012 to October 2013. Galls were field-collected with either late-instar larvae or pupae inside, on shoots of *Tibouchina sellowiana* (Cham.) Cogn. (São Francisco de Paula, RS), *Tibouchina asperior* (Cham.) Cogn. (Santo Antônio da Patrulha, RS) and *Tibouchina fissinervia* (Schrank & Mart. ex DC.) Cogn. (Camacan, Bahia). Immature stages were obtained by dissecting additional galls. Adult specimens were pinned and dry preserved. The immatures were fixed in Kahle-Dietrich´s fluid and preserved in 75% EtOH. For DNA analyses, additional specimens were preserved in 100% EtOH at -20 °C.

For gross morphology studies, the specimens were cleared in a 10% potassium hydroxide (KOH) solution and mounted on slides with either glycerin jelly or Canada balsam. Observations were made with the aid of a Leica® M125 stereomicroscope. Structures selected to be drawn were previously photographed with an attached Sony® Cyber-shot DSC-H10 digital camera. Then, vectorized line drawings were made with the software CorelPhotoPaint® X4, using the corresponding digitalized images as a guide. At least five specimens were used for the descriptions of each life stage. Measurements were made with an attached ocular micrometer; values are presented as mean ± standard deviation unless noted otherwise.

Specimens used in scanning electron microscope (SEM) analyses were dehydrated in a Bal-tec® CPD030 critical-point dryer, mounted with double-sided tape on metal stubs, and coated with gold in a Bal-tec® SCD050 sputter coater. They were examined and photographed in a JEOL® JSM5800 scanning electron microscope at Centro de Microscopia Eletrônica (CME) of UFRGS.

**Molecular phylogeny.** Total genomic DNA was purified from larval tissue, using a Qiagen DNA Blood and Tissue Kit, to investigate: (i) monophyly of *Palaeomystella fernandesi*, *Palaeomystella rosaemariae* and *Palaeomystella tavaresi*; and (ii) reconstruct phylogenetic relationships within *Palaeomystella*. For comparison, two pupae of *Palaeomystella oligophaga* Becker & Adamski, 2008, from a population of *Macairea radula* (Bonpl.) DC. located in Brasília, Distrito Federal, were also used for DNA extraction (Table 1). Of the mitochondrial gene cytochrome *c* oxidase subunit I (CO-I) a piece of 660 base pairs (bp) was amplified using the universal primers LCO1490 (5'-ggtcaacaaatcataaagatattgg-3') and HCO2198 (5'-taaacttcagggtgaccaaaaaatca-3') and following PCR conditions proposed by Folmer et al. (1994). PCR products were treated with Exonuclease I and FastAP™ Thermosensitive Alkaline Phosphatase (Thermo Scientific), sequenced using the BigDye® chemistry, and analyzed on an ABI3730XL DNA analyzer (Applied Biosystems Inc.) at Macrogen (Seoul, Republic of Korea). Sequences were aligned and visually inspected using the algorithm Clustal X in MEGA 5 (Tamura et al. 2011) running in full mode with no manual adjustment. All data generated in this study were deposited in GenBank under the accession numbers KJ188233–KJ188246 (Table 1) and BOLD system, under the project "Momphidae of Brazil" (MOMBR001-14 to 014-14). A phylogenetic tree was reconstructed in order to test the proposed hypothesis of monophyletic status for the three members of *Palaeomystella*: *Palaeomystella fernandesi*, *Palaeomystella rosaemariae* and *Palaeomystella tavaresi*. The internal relationships of these taxa within *Paleomystella* and with other species were also investigated. The single currently recognized and named taxon (*Palaeomystella oligophaga*) as well as undescribed species (*Palaeomystella* sp. 1 and *Palaeomystella* sp. 2) were used in order to cover the widest possible diversity of the genus. Accordingly, variants that match exactly the previously sequenced region in a representative taxon of the sister group of Momphidae (genus *Mompha* Hübner, 1825) were obtained from GenBank and were incorporated into our analysis as the outgroup (Table 1).

**Table 1.** Specimens used in this study to reconstruct the phylogenetic relationships of the new species of *Paleomystella*, based on cytochrome oxidase subunit I sequences.

Genus	Species	Voucher	GenBank accession numbers
**Ingroup**			
*Palaeomystella*	*Palaeomystella oligophaga*	LMCI 234-1A	KJ188233
		LMCI 234-1B	KJ188234
	*Palaeomystella* sp. 1	LMCI 211-4A	KJ188235
		LMCI 211-4B	KJ188236
	*Palaeomystella* sp. 2	LMCI 174-25	KJ188237
		LMCI 174-26	KJ188238
		LMCI 174-27	KJ188239
	*Palaeomystella tavaresi* sp. n.	LMCI 209-6A	KJ188240
		LMCI 209-6B	KJ188241
	*Palaeomystella rosaemariae* sp. n.	LMCI 211-8A	KJ188242
		LMCI 211-8B	KJ188243
	*Palaeomystella fernandesi* sp. n.	LMCI 174-50A	KJ188244
		LMCI 174-50B	KJ188245
		LMCI 174-56	KJ188246
**Outgroup**			
*Mompha*	*Mompha conturbatella*	10-JDWBC-1043	HM862677

Phylogenetic reconstructions were based on two methods: Bayesian inference (BI), implemented in BEAST 2.0 (Drummond et al. 2012) and maximum likelihood (ML), run in PHYML 3.0 (Guindon et al. 2010). In BI, a relaxed uncorrelated lognormal clock was used together with no fixed mean substitution rate and a Yule prior on branching rates, using the GTR [General Time-Reversible] (Rodríguez et al. 1990) model of sequence evolution. Four independent runs were used, of 10 million generations and a burn-in period of 100, 000 (the first 1000 trees were discarded); the remaining trees were summarized in TreeAnnotator 1.6.2 (Drummond and Rambaut 2007) and used to infer a maximum a posteriori consensus tree. Posterior probabilities were used as an estimate of branch support. For ML, the program jModeltest (Posada 2008) was used to estimate the substitution model GTR + G, with gamma distribution (G) according to the Akaike Information Criterion. Monophyly-confidence limits were assessed with the bootstrap method (Felsenstein 1985) at 60% cut-off after 1000 bootstrap iterations. Trees were inspected and edited in FigTree 1.3.1 (http://tree.bio.ed.ac.uk/software/figtree). The evolutionary distance using the Kimura 2-parameters (K2P) model (Kimura 1980) procedure, with 1000 bootstrap replications, was analyzed between groups, as follows: 1) outgroup *Mompha conturbatella* (Hübner, 1819); 2) *Palaeomystella* sp. 1; 3) *Palaeomystella* sp. 2; 4) *Palaeomystella fernandesi* sp. n.; 5) *Palaeomystella tavaresi* sp. n.; 6) *Palaeomystella rosaemariae* sp. n.; 7) *Palaeomystella oligophaga*.

### Museum collections

Abbreviations of the Brazilian states and institutions from which specimens were examined are:

BA Bahia State.

DF Distrito Federal.

DZUP Coll. Padre Jesus S. Moure, Departamento de Zoologia, Universidade Federal do Paraná, Curitiba, Paraná.

LMCI Laboratório de Morfologia e Comportamento de Insetos, Universidade Federal do Rio Grande do Sul, Porto Alegre, Rio Grande do Sul.

MCTP Museu de Ciências e Tecnologia da Pontifícia Universidade Católica do Rio Grande do Sul, Porto Alegre, Rio Grande do Sul.

RS Rio Grande do Sul State.

VOB Coll. Vitor O. Becker, Reserva Serra Bonita, Camacan, Bahia.

## Results

### Palaeomystella fernandesi

Taxon classificationAnimaliaLepidopteraMomphidae

Moreira & Becker sp. n.

http://zoobank.org/F2B43BAC-11CB-4374-B6C4-17E8CB82C8DC

**Figure 1.**
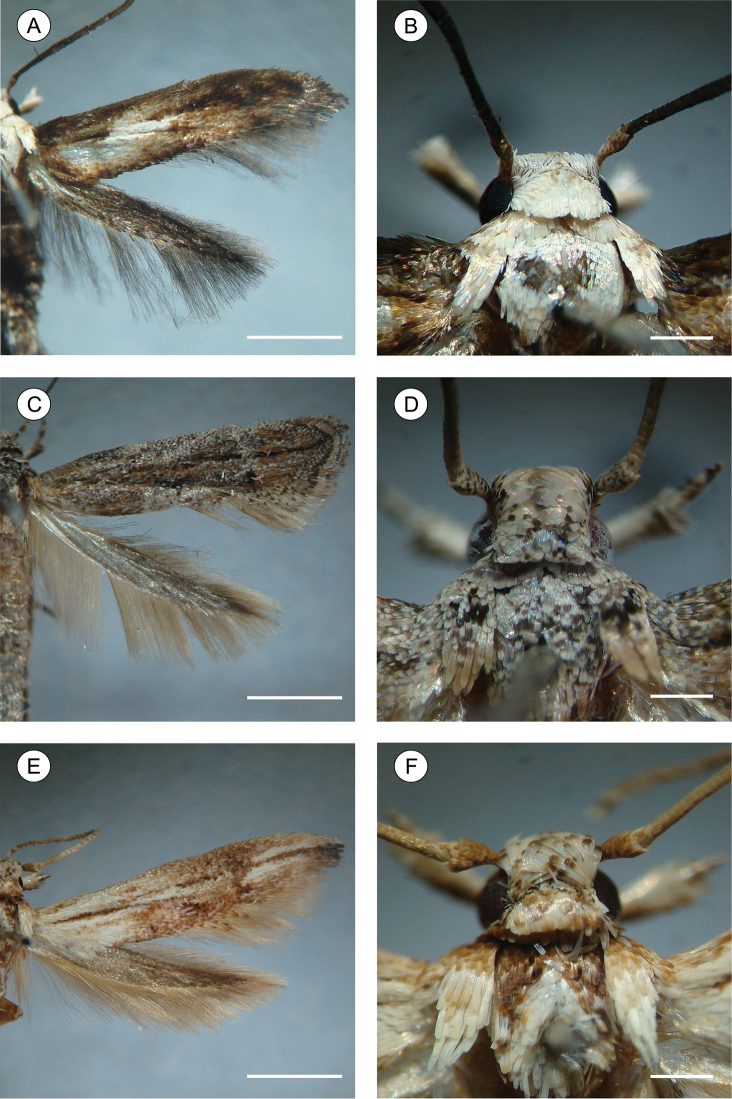
Spread right wings (left column), head and thorax in detail (right column) of pinned *Palaeomystella* species, dorsal view: **A–B** *Palaeomystella fernandesi* **C–D** *Palaeomystella rosaemariae* **E–F** *Palaeomystella tavaresi*. Scale bars = 2, 0.5, 2, 0.5, 2 and 0.5 mm, respectively.

**Figure 2.**
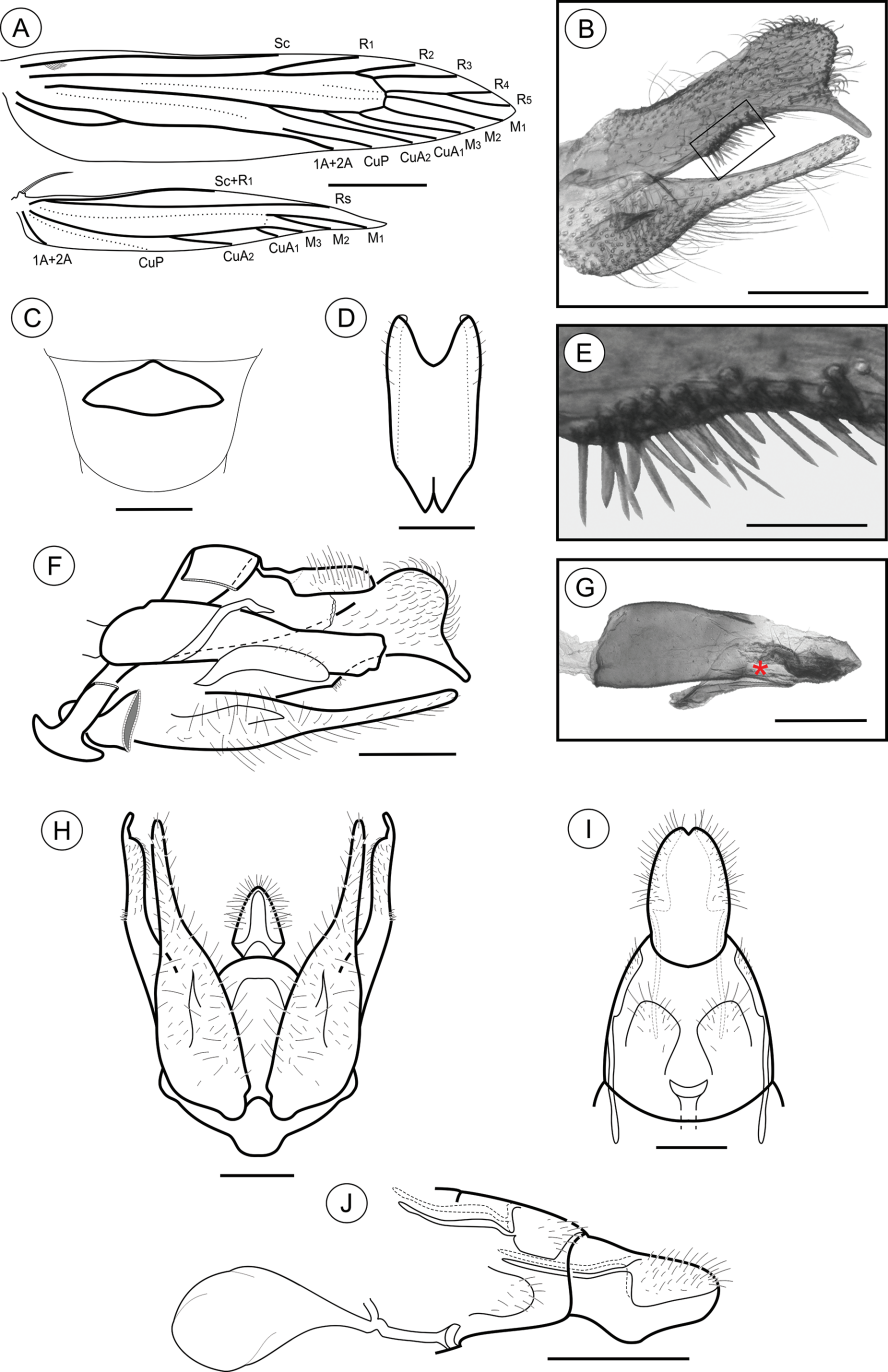
*Palaeomystella fernandesi* adult morphology: **A** wings **B** male valva, mesolateral view **C** male eighth sternum, ventral view **D** juxta, ventral **E** ventral spines of the valva upper section in detail (rectangular area shown in **B**), mesolateral view **F** male genitalia, lateral view **G** aedeagus, lateral view (asterisk indicates attached juxta-lobes) **H** male genitalia, ventral view (transtilla, aedeagus and juxta not illustrated) **I** female genitalia, ventral view (corpus bursae not illutrated) **J** female genitalia, lateral view. Scale bars = 1 mm; 200, 200, 100, 50, 200, 200, 200 and 250µm; 0.5 mm, respectively.

**Figure 3.**
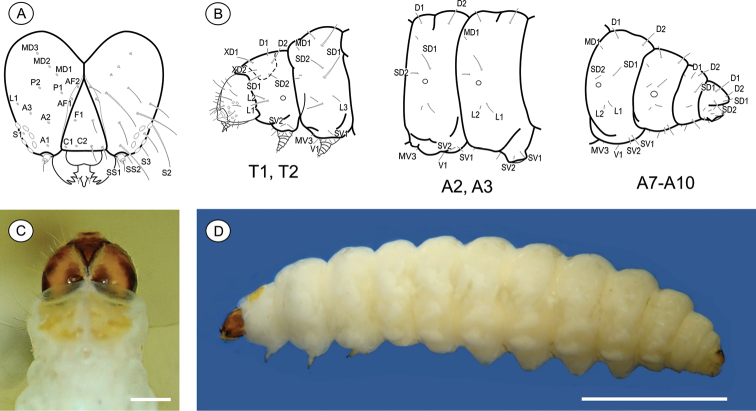
*Palaeomystella fernandesi* last larval instar: **A** cephalic chaetotaxy, frontal view **B** thoracic and abdominal chaetotaxy, lateral view **C** head and prothoracic shield in detail, dorsal view **D** body, lateral view. Scale bars = 50 µm, 1 mm, respectively.

**Figure 4.**
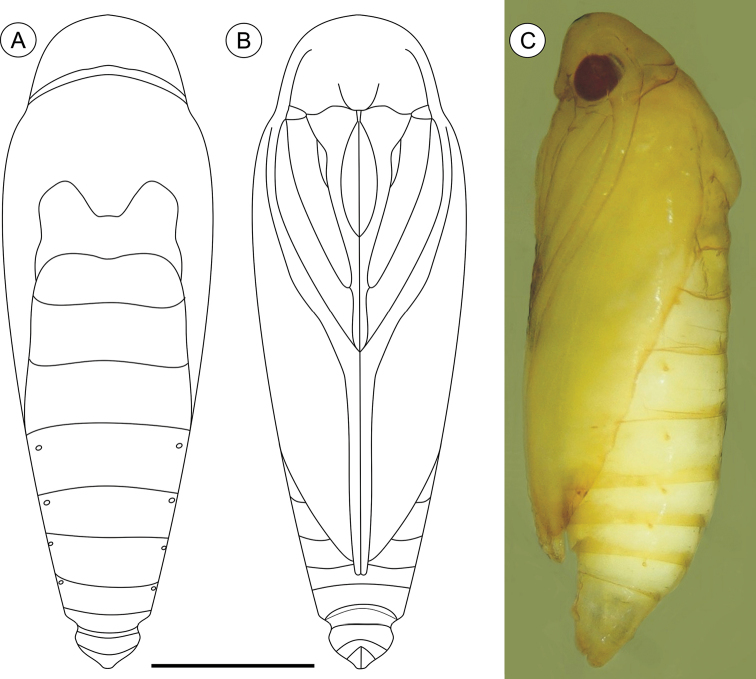
*Palaeomystella fernandesi* pupa, in dorsal (**A**), ventral (**B**) and lateral (**C**) views, respectively. Scale bar = 1 mm.

Figs 1A–B , 2 – 4 , 11A–C , 12A–C

#### Diagnosis.

Although showing congeneric affinity, *Palaeomystella fernandesi* has morphological features that in conjunction distinguish it from all known *Palaeomystella* species, as follows: 1) male genitalia with upper section of valve narrowing distally, forming a single process that bends medially; 2) pupa with cremaster short and apically rounded, with four pairs of setae; 3) galls of fusiform type, external surface without conspicuous ornament, bearing a few longitudinal carinae, induced on stem of *Tibouchina sellowiana* apical branches.

#### Description.

**Adult** (Figs 1A–B). Sexes similar in size and color; Forewing length 4.68 to 6.11 mm (n = 7). *Head* (Fig. 1B): Frons and vertex creamy white; labial palpus mostly dark brown, basal segments angled laterally, terminal segment slightly angled upward; antennae dark brown; proboscis yellowish brown. *Thorax*: Tegula and mesonotum whitish creamy white with pale-brown scales; legs dark brown. Forewing (Figs 1A, 2A): lanceolate, with 13 veins; L/W index ~ 5.1; dorsally covered mostly by dark-brown scales; with three interconnected white areas that form a longitudinal S-like band; one proximal, rounded, in the anal area, made of pale-creamy white scales, followed by a short stripe aligned in the cubital area, made of creamy white scales, and a third, also rounded and faint, in the cell, made of pale-creamy white scales; a tenuous, U-shaped band of pale-gray scales following the contours of the tornus; three raised tufts of pale-gray scales, located posteriorly to cubitus, in anal area, in line with mid-cell, and near tornal area respectively; fringes dark brown; ventrally mostly covered by dark-brown scales; retinaculum subcostal; discal cell closed, ~ 0.8× length of forewing, ending near 1/5 of wing margin; Sc ending ca. middle of anterior margin; R 5-branched; R_1_ ending near 1/3 of wing margin; R_4_ and R_5_ stalked ca. 1/2 distance from the cell apex; M 3-branched; CuA 2-branched; CuP weak proximally and not stalked, with well-developed 1A+2A extending more than 1/2 posterior margin. Hindwing (Figs 1A, 2A): strongly lanceolate, with nine veins; L/W index ~ 7.2, ~ 0.8 forewing in length; scales dark brown on both sides; fringes dark brown; frenulum a single acanthus in male, with two distally directed acanthi in female; Sc+R_1_ ending ca. 1/2 anterior margin; Rs ending ca. 1/5 anterior margin; M 3-branched, M_1_ and M_2_ stalked from remnant chorda of cell, from point beyond base of Rs; CuA 2-branched, with CuA_1_ stalked to M3; CuP weakly sclerotized, ending 1/3 posterior margin; 1A+2A well developed, ending near basis of posterior margin*. Abdomen* (not illustrated): pale brown, intermixed with gray scales, with transverse irregular rows of spiniform setae on terga 2–7 in both sexes; eighth sternum (Fig. 2C) anteriorly expanded medially into a short lobe, associated with a subtriangular sternite.

**Male genitalia** (Figs 2B, D–H). Uncus narrow, separated from tegumen by a narrow membranous area, distally roof like and laterally setose (Figs 2F, H); tegumen narrow; vinculum widened ventrally; transtilla a flat, rounded fig; aedeagus cylindrical, moderately long, slightly wider basally (Fig. 2G); vesica bearing a few short stout cornuti; juxta (Fig. 2D) attached to distal portion of aedeagus (Fig. 2G), longer than wide, with two small, parallel pointed projections mid-anteriorly and deeply concave distally; valva (Figs 2B, F, H) covered with several long setae, divided near 1/3 from the basis, with a long, finger-like sacculus and a wide spatulate costa, bearing a thin ventral finger-like projection near apex, and several stout, medium-sized spines meso-ventrally (Fig. 2E).

**Female genitalia** (Figs 2I–J). Papillae anales connected dorsally, narrowed distally, setose; anterior apophyses with arms slightly curved, similar in length to posterior apophyses; sterigma divided into a bandlike tergum and a distally bilobed sternum, shallowly and widely emarginate medially; ostium bursae small, wider than long; ductus bursae membranous, shorter than corpus bursae; ductus seminalis inserted distally; corpus bursae an elongate sac, with no sclerotizations on inner wall.

#### Type material.

Holotype ♂ **Brazil:** Centro de Pesquisas e Conservação da Natureza Pró-Mata (CPCN Pró-Mata; 29°29'16"S, 50°10'60"W; 925 m), São Francisco de Paula, RS, Brazil. Dry preserved pinned adults, reared from galls induced on *Tibouchina sellowiana* (Cham.) Cogn. (Melastomataceae), LMCI 210-56, 7–9.III.2013, by G.R.P. Moreira, F.A. Luz and L.T. Pereira, donated to DZUP (29.409). **Paratypes:** same data, 26.III.2012, by G.R.P. Moreira, F.A. Luz and P. Pollo; 2♀ (LMCI 174-161 and 162), donated to DZUP (29.410 and 29.411); 1♂ (LMCI 174-157) with genitalia in glycerin (GRPM 50-51) and 1♀ (LMCI 174-158), donated to MCTP (36.225 and 36.226, respectively).

#### Other specimens examined.

With the same collection data, deposited in LMCI. Adults, dried and pinned: 2♂ (LMCI 174-159 and 210-49), 1♀ (LMCI 174-160), 1♀ (LMCI 174-163) with genitalia in glycerin (GRPM 50-52). Adults, fixed in Kahle-Dietrich’s fluid and preserved in 70% EtOH: 1♂ (LMCI 174-165), 3♀ (LMCI 174-164, 166 and 167). Slide preparations, mounted in Canada balsam: genitalia, 3♂ (GRPM 50-29, 47 and 48), 1♀ (GRPM 50-28); wings, 2 ♂ (GRPM 50-45 and 50), 1♀ (GRPM 50-46); larvae, 2 last instars (GRPM 50-49). Immature stages, fixed in Kahle-Dietrich’s fluid and preserved in 70% EtOH: 8 last-instar larvae (LCMI 174-52); 7 pupae (LMCI 174-168, 169 and 223; and 210-16); 10 galls (LMCI 174-47 to 49, 174-217 to 222, and 210-15). In tissue collection, 9 larvae (LMCI 174-50 and 56) fixed and preserved in 100% EtOH, at -20°C.

#### Immature stages.

**Last instar larva** (Figs 3A–D), 3.51 to 7.01 mm (n = 6). Cecidogenous, endophyllous, semiprognathous, and tissue-feeder. Body with setae well developed. *Head* (Figs 3A, C–D): brown, with two paler mid-dorsal areas; smooth, with shallow ridges; labrum shallowly notched; frons higher than wide, extending ca. 3/4 epicranial notch; six stemmata arranged in C-shape. Chaetotaxy (Fig. 3A): A-group trisetose; L-group unisetose; P-group bisetose; MD trisetose; C-group bisetose; F-group unisetose; AF-group bisetose; S-group trisetose; SS-group trisetose. A1, A3, P1 and S2 about equal in length, longest setae on head; C1, C2, F1, A2, AF2, L1 intermediate in length; AF1 shorter; MD1–3 very reduced and aligned with each other. Antenna two-segmented. Mandibles broad with four teeth, and one seta on outer surface; labium broad, with two-segmented palpus and spinneret parallel-sided; maxilla prominent. *Thorax and abdomen* (Figs 3B–D): Prothoracic shield light brown, divided longitudinally by indistinctly marked, unpigmented area; anal fig brown. Thoracic legs slightly pigmented. Prolegs on A3–A6 and A10 of equal size; crochets in a circle, uniserial and uniordinal. Thorax chaetotaxy: T1 with D-group bisetose, both located on the dorsal shield, D1 shorter than D2; XD-group bisetose, setae similar in length and both on the dorsal shield; SD bisetose, laterally on the dorsal shield; L-group bisetose, L1 longer than L2; SV-group bisetose, posteroventral to L2, SV1 slightly longer than SV2; V-group unisetose. T2 and T3 with D- and SD-groups bisetose, median-transversely aligned; D2 and SD1 similar in length, and longer than D1 and SD2 respectively; L trisetose, L3 posterior to L1–L2, similar in length to L1; SV unisetose; V unisetose. Abdomen chaetotaxy: D-group bisetose; A1–9 with D2 slightly longer than D1, and A10 with D1 longer than D2; SD-group bisetose, A1–7 with SD1 slightly longer than SD2 and A10 with SD2 longer than SD1, SD2 absent in A9; A1–8 with L-group bisetose, L1 longer than L2, L2 absent in A9; A1–8 with SV-group bisetose, SV1 slightly shorter than SV2, SV1 absent in A9; V-group unisetose.

**Pupa** (Figs 4A–C, 11A–C), 4.42 to 6.11 mm long (n = 5). Body elongate-oval in dorsal and ventral views, widest and dorsally raised in mesothoracic region. Integument weakly melanized, mostly smooth, with a few scattered microsetae dorsally. Frontoclypeal suture not evident. Labrum U-shaped. Labial palpi long; antennae arched anteriorly and separate, approximate and parallel posteriorly to distal margins of maxillae, surpassing apical margin of forewings; maxillae extending distally between sclerites of mid-legs; femora of midleg not fused distally; femora of foreleg extending beyond widest part of labial palpi. Cremaster (Figs 11A–C) short and apically rounded, with four pairs of setae; one latero-basally, another latero-dorsally and two latero-distally.

#### Distribution.

Known only from the type locality, in the Dense Umbrophilous Forest (= Brazilian Atlantic Rain Forest *sensu stricto*) portions of the CPCN Pró-Mata, São Francisco de Paula, RS, Brazil.

#### Host Plant.

*Tibouchina sellowiana* (Cham.) Cogn. (Melastomataceae). A small tree (3 to 6 m), endemic to the coastal montane forests of southern Brazil, ranging from Minas Gerais to Rio Grande do Sul, usually flowering in April–May (Souza 1986, Guimarães 2014).

#### Life history

(Figs 12A–C). Galls induced by *Palaeomystella fernandesi* are common on *Tibouchina sellowiana* at the type locality, during spring (October) and summer (February). They are prosoplasmatic histioid (Küster, in Meyer 1987); fusiform, 6.0 to 18 mm long (n = 12); induced on stem apex (Fig. 12A); without conspicuous projections, bearing a few longitudinal carinae on surface and changing gradually from green to violet as ages; fleshy, without uniformly defined internal chamber; unilocular, unilarval. Most of them house a specialized kleptoparasitic gelechiid moth, whose complex natural history is described in detail elsewhere (Luz et al. 2014). Those that are free from the kleptoparasite fall to the ground in late larval ontogeny and larva complete development on the ground. Pupation occurs inside the gall, within a cylindrical, longitudinally arranged cocoon made of woven white silk (Fig. 12C). The adult emerges presumably after the winter (September), through a circular operculum (with plant epidermis and penellipse white silk frill) on upper half of gall wall (Fig. 12B) constructed by the last instar larva prior to its pupation.

#### Etymology.

Named in honor of Prof. Dr. Geraldo Wilson Fernandes, Departamento de Biologia Geral, Instituto de Ciências Biológicas, Universidade Federal de Minas Gerais, for his great contributions to the development of cecidology in the Neotropics.

### Palaeomystella rosaemariae

Taxon classificationAnimaliaLepidopteraMomphidae

Moreira & Becker sp. n.

http://zoobank.org/02056832-C637-4552-8A83-2F896EEE4143

**Figure 5.**
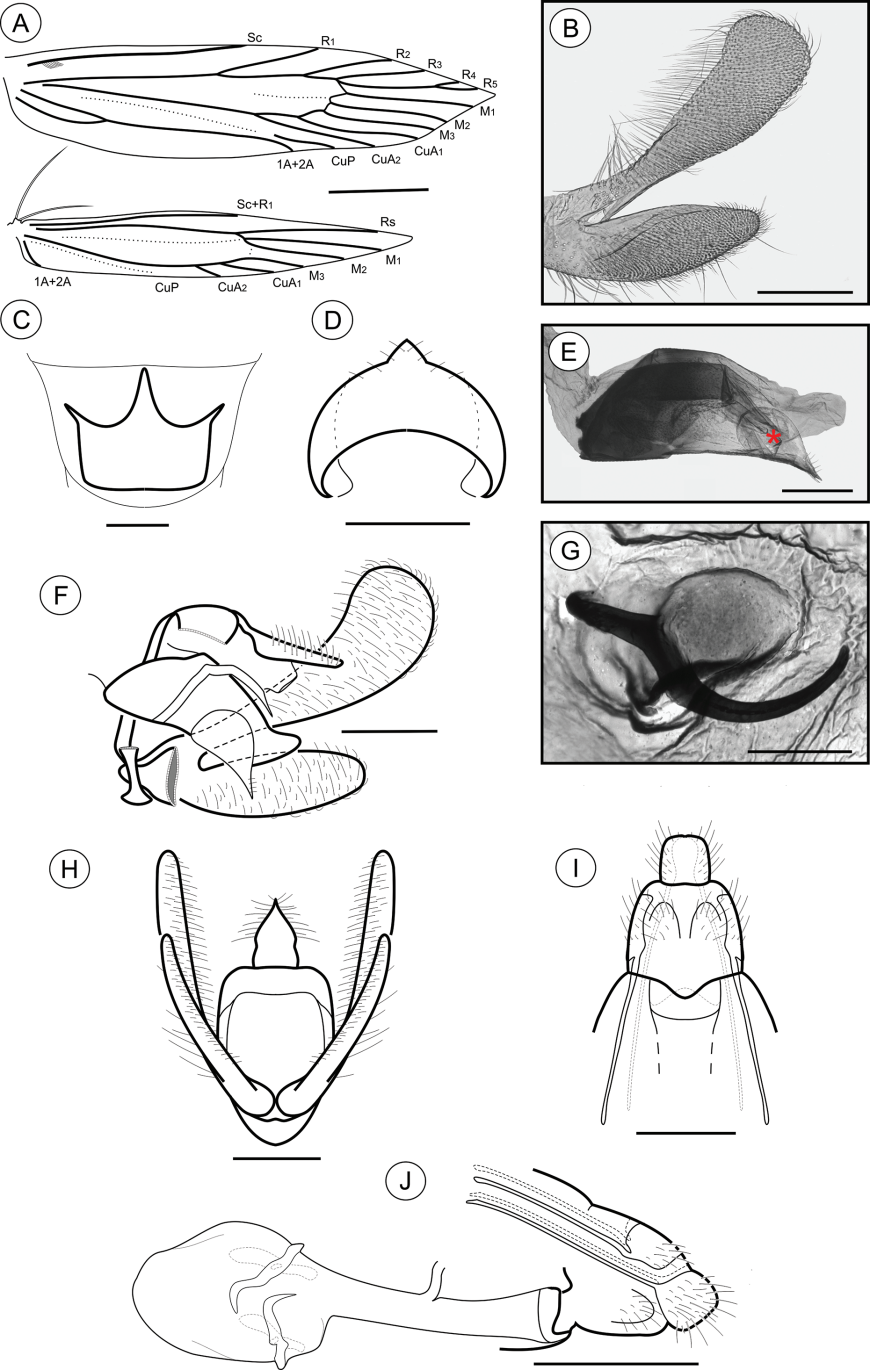
*Palaeomystella rosaemariae* adult morphology: **A** wings **B** male valva, mesolateral view **C** male eighth sternum, ventral view **D** juxta, ventral; **E** aedeagus, lateral view (asterisk indicates attached juxta-lobes) **F** male genitalia, lateral view **G** signum, internal view **H** male genitalia, ventral view (transtilla, aedeagus and juxta not illustrated) **I** female genitalia, ventral view (corpus bursae not illustrated) **J** female genitalia, lateral view. Scale bars = 1 mm; 200, 200, 100, 200, 100, 200, 200 and 250 µm; 0.5 mm, respectively.

**Figure 6.**
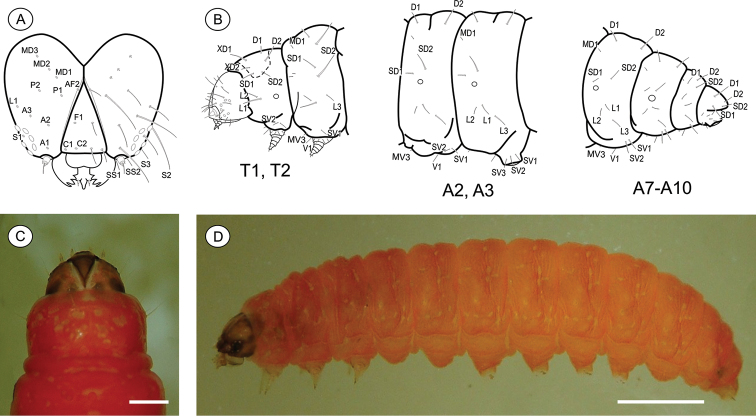
*Palaeomystella rosaemariae* last larval instar: **A** cephalic chaetotaxy, frontal view **B** thoracic and abdominal chaetotaxy, lateral view **C** head and prothoracic shield in detail, dorsal view **D** body, lateral view. Scale bars = 50 µm, 1 mm, respectively.

**Figure 7.**
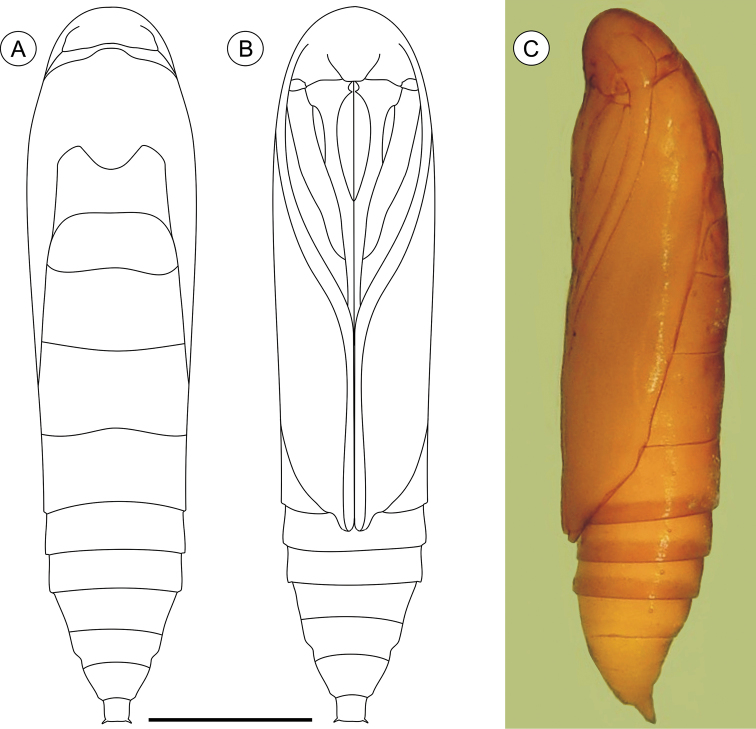
*Palaeomystella rosaemariae* pupa, in dorsal (**A**), ventral (**B**) and lateral (**C**) views, respectively. Scale bar = 1 mm.

Figs 1C–D , 5 – 7 , 11D–F , 12D–F

#### Diagnosis.

Closest to *Palaeomystella tavaresi*, sharing with this species a valve with a pronounced palmate costa and bladelike signa. These characters distinguish them from all other species of *Palaeomystella* except *Palaeomystella oligophaga*. This, however, has the forewings with R_4_-R_5_ fused and the hindwing with M_1_ and M_2_ stalked from the remnant chorda of the cell (Becker and Adamski 2008). *Palaeomystella rosaemariae* differs from *Palaeomystella tavaresi* by having: 1) adults, body covered with pale-brown scales interspersed with pale-brown scales tipped with dark brown; 2) males with latero-anterior margin of eighth sternite deeply concave; upper distal section of valva narrower; juxta as long as wide, slightly concave anteriorly; 3) females with signa with inward projection long, thin and curved; 4) pupa with cremaster tubular, dorsally directed, bearing latero-apically a pair of anteriorly curved spines; 5) galls globose, with external surface covered with short spine-like projections, induced on terminal buds of *Tibouchina asperior*.

#### Description.

**Adult** (Figs 1C–D). Sexes similar, forewing length 4.81 to 5.59 mm (n = 5). *Head* (Fig. 1D): Frons pale brown; vertex and labial palpus and antenna with pale-brown scales tipped with dark brown; labial palpus with basal segments angled laterally, terminal segment slightly angled upward; proboscis yellowish brown. *Thorax*: Tegula and mesonotum with pale-brown scales tipped with dark brown, posterior scales having more pale brown; fore and midlegs dark brown; hindlegs pale brown, tibia and tarsus with intermixed dark-brown scales. Forewing (Figs 1C, 5A): lanceolate, with 13 veins; L/W index ~ 4.5; dorsally covered by pale-brown scales intermixed with scattered, pale-brown scales tipped with dark brown, and with longitudinally aligned groups of brown scales; a narrow, ill-defined, dark-brown streak bisecting the wing longitudinally from base to tornus; 3 raised scale tufts located posterior to cubitus, including 1 wider tuft in anal area, 1 in line with midcell, and 1 near tornal area; fringes pale brown, interspersed with a few pale-brown scales tipped with dark brown; tornal area with two bands of pale-brown scales tipped with blackish brown; ventrally, mostly uniformly covered with dark-brown scales; retinaculum subcostal; discal cell closed, ~ 2/3 length of forewing; ending near 1/5 of wing margin; Sc ending ca. middle of anterior margin; R 5-branched; R_1_ ending near 1/3 of wing margin; R_4_ and R_5_ stalked ca. 1/4 distance from the cell apex; M 3-branched; CuA 2-branched; CuP weak proximally and not stalked, with 1A+2A that is well developed, extending more than half length of posterior margin. Hindwing (Fig. 5A) strongly lanceolate, with 9 veins; L/W index ~ 6.4, ~ 0.8 forewing in length; scales pale brown on both sides; fringes pale brown; frenulum with a single acanthus in male, and with two acanthi in female, proximal acanthus anteriorly divergent, and distal acanthus parallel to wing anterior margin; Sc+R_1_ ending at ca. 1/2 anterior margin; Rs ending at ca. 1/5 anterior margin; M 3-branched; CuA 2-branched, with CuA_1_ stalked to M3; CuP weakly sclerotized, ending at 1/3 posterior margin; 1A+2A well developed, ending near basis of posterior margin. *Abdomen* (not illustrated): pale-brown scales intermixed with gray scales, with transverse irregular rows of spiniform setae on terga 2–7 in both sexes; eighth sternum (Fig. 5C) anteriorly expanded medially into a slender, sharply pointed lobe, associated with a subtrapezoidal sternite.

**Male genitalia** (Figs 5B, D–F, H). Uncus narrow, separated from tegumen by a narrow membranous area, laterally setose (Fig. 5F); tegumen narrow, widened dorsally; vinculum widened ventrally; transtilla a short, flat fig; aedeagus tubiform, short (twice as long as wide), curved ventrally, slightly wider basally (Figs 5E–F); vesica bearing several stout cornuti; juxta (Fig. 5D) attached to distal portion of aedeagus (Fig. 5E), wider than long, with slightly concave anterior margin and pointed distally; valva (Figs 5B, F, H) covered with several long setae, divided near 1/3 from base, with flat, broad sacculus tapering distad, and long, spatulate costa, rounded distally and gradually constricted toward base.

**Female genitalia** (Figs 5G, I–J). Papillae anales connected dorsally, setose (Figs 5I–J); anterior apophyses slightly shorter than posterior apophyses; sterigma divided into a bandlike tergum and a distally bilobed sternum, shallowly emarginate medially; ostium bursae large, wider than long; ductus bursae membranous, longer than corpus bursae; ductus seminalis inserted medially; corpus bursae an elongate sac, bearing two narrow and curved, bladelike signa that are connected to transversely elongate, rounded figs located on the wall (Figs 5G, J).

#### Type material.

**Holotype** ♂: **Brazil:** Private farm belonging to Antonio Malta, Coxilha das Lombas, 30°02'13"S, 50°36'30"W, 17 m, Santo Antônio da Patrulha, RS, Brazil. Dry preserved pinned adults, reared from galls induced on *Tibouchina asperior* (Cham.) Cogn. (Melastomataceae), LMCI 211, 12.III.2013, by G.R.P. Moreira, F.A. Luz and S. Bordignon, (LMCI 211-12), donated to DZUP (29.412). **Paratypes:** same data, 1♂, 1♀ (LMCI 211-14 and 06) with genitalia in glycerin (GRPM 50-43 and 44), donated to DZUP (29.413 and 29.414, respectively).

#### Other specimens examined.

Dry preserved pinned adults, with the same collection data, deposited in LMCI under the following accession numbers: 2♂ (LMCI 211-07 and 10); 1♀ (LMCI 211-11). Slide preparations, mounted in Canada balsam: genitalia, 2♂ (GRPM 50-38 and 39), 1♀ (GRPM 50-40); wings, 1♂ (GRPM 50-36), 1♀ (GRPM 50-37); larvae, 2 last instars (GRPM 50-41 and 42). Immature stages, fixed in Kahle-Dietrich’s fluid and preserved in 70% EtOH: 6 last-instar larvae (LCMI 211-17 to 22); 3 pupae (LMCI 211-5, 9 and 26); 6 mature, intact galls (LMCI 211-25). In tissue collection, 6 larvae (LMCI 211-8), fixed and preserved in 100% ethanol, at -20°C.

#### Immature stages.

**Last-instar larva** (Figs 6A–D), 4.94 to 9.88 mm long (n = 5). Cecidogenous, endophyllous except prior to pupation, semiprognathous and tissue-feeder. Body subcylindrical, creamy white, changing to red when mature prior to exit the gall; with setae well developed. *Head* (Figs 6A, C–D): pale brown, interspersed with two pairs of darker mid-dorsal areas; smooth, with shallow ridges; labrum shallowly notched; frons higher than wide, extending ca. 3/4 epicranial notch; six stemmata arranged in C-shaped configuration. Chaetotaxy (Fig. 6A): A-group trisetose; L-group unisetose; P group bisetose; MD trisetose; C group bisetose; F group unisetose; AF group bisetose; S group trisetose; SS group trisetose. A1, A3, P1 and S2 about equal in length, longest setae on head; C1, C2, F1, A2, AF2, L1 intermediate in length; AF1 absent; MD1–3 very reduced and aligned with each other. Antenna two-segmented. Mandibles broad with four teeth, and one seta on the outer surface; labium broad, with two-segmented palpus, the distal segment minute; spinneret parallel-sided; maxilla prominent. *Thorax and Abdomen* (Figs 6B–D): Prothoracic shield and anal fig slightly marked by irregularly shaped, small light-brown blots. Thoracic legs also scarcely pigmented. Prolegs on A3-A6 and A10 of equal size; crochets in a semicircle, uniserial and uniordinal. Thorax chaetotaxy: T1 with D group bisetose, both located on dorsal shield, D1 shorter than D2; XD group bisetose, similar in length and both on the dorsal shield; SD bisetose, laterally on the dorsal shield; L group bisetose, L1 longer than L2; SV group bisetose, posteroventral to L2, SV1 slightly longer than SV2; V group unisetose. T2 and T3 with D and SD groups bisetose, median-transversely aligned; D2 and SD1 similar in length, and longer than D1 and SD2 respectively; L trisetose, L3 posteriorly, similar in length to L1; SV unisetose; V unisetose. Abdomen chaetotaxy: D group bisetose; A1–9 with D2 slightly longer than D1, and A10 with D1 longer than D2; SD group bisetose, A1–7 with SD1 slightly longer than SD2 and A10 with SD2 longer than SD1, SD2 absent in A9; A1–8 with L group trisetose, L1 longer than L2, L1 and L2 absent in A9; A1–8 with SV group trisetose, SV3 absent in A7–9; V group unisetose.

**Pupa** (Figs 7A–C, 11D–F), 5.59 to 6.76 mm long (n = 3), elongate in dorsal and ventral views, slightly wider in thoracic region. Integument light amber-colored, mostly smooth, with a few scattered microsetae dorsally. Frontoclypeal suture not evident. Labrum U-shaped. Labial palpi long; antennae arched anteriorly and separate, approximate and parallel posteriorly to distal margins of maxillae, reaching apical margin of forewings; maxillae extending distally between sclerites of midlegs; femora of midleg not fused distally; femora of foreleg extending beyond widest part of labial palpi. Cremaster (Figs 11D–F) long, tubular, dorsally directed, bearing latero-apically a pair of distally conspicuous, anteriorly curved spines.

#### Distribution.

*Palaeomystella rosaemariae* is known only from the type locality, the fragments of lowland Dense Umbrophilous Atlantic Forest of Coxilha das Lombas, Santo Antônio da Patrulha, RS, Brazil.

#### Host plant.

*Tibouchina asperior* (Cham.) Cogn. (Melastomataceae), a shrub (0.5 to 1.0 m), in humid grassland areas, endemic to Santa Catarina and Rio Grande do Sul (Souza 1986, Guimarães 2014). At Coxilha das Lombas, where the southernmost portions of lowland Dense Umbrophilous Atlantic Forest occurs, these shrubs are common along the borders of forest fragments located in poorly drained, swampy areas, associated with the formation of lagoons and also influenced by sand dunes.

#### Life history

(Figs 12D–F). Galls induced by *Palaeomystella rosaemariae* are located at distal axillary buds of the host. At the type locality, they occur in low numbers per plant. Galls are prosoplasmatic histioid (Küster, in Meyer 1987); small, delicate, globoid (5.2 to 7.28 mm long; n = 7), green to reddish, covered with several short spine-like projections (Fig. 12D). Unilocular, unilarval, pupates away from the gall. Little is known about the life history of this species. In laboratory, mature last instar larva invariably made a lateral orifice by chewing the gall wall (Fig. 12E) and moved directly to the bottom of the plastic pot. There, they promptly began to construct a cocoon by tying together small pieces of dried leaves with silk, where the pupation occurred (Fig. 12F). The adult emerged through a slit made at the terminal end of the cocoon. Specimens that pupated in the laboratory during the summer emerged as adults in the following autumn (May).

#### Etymology.

Named in honor of Prof. Dr. Rosy Mary dos Santos Isaias, an anatomist of the Departamento de Botânica, Instituto de Ciências Biológicas, Universidade Federal de Minas Gerais, for her great contributions to the development of cecidology in the Neotropics.

### Palaeomystella tavaresi

Taxon classificationAnimaliaLepidopteraMomphidae

Becker & Moreira sp. n.

http://zoobank.org/D4D1FE46-9C47-4F6B-B4D4-D981BC83F577

**Figure 8.**
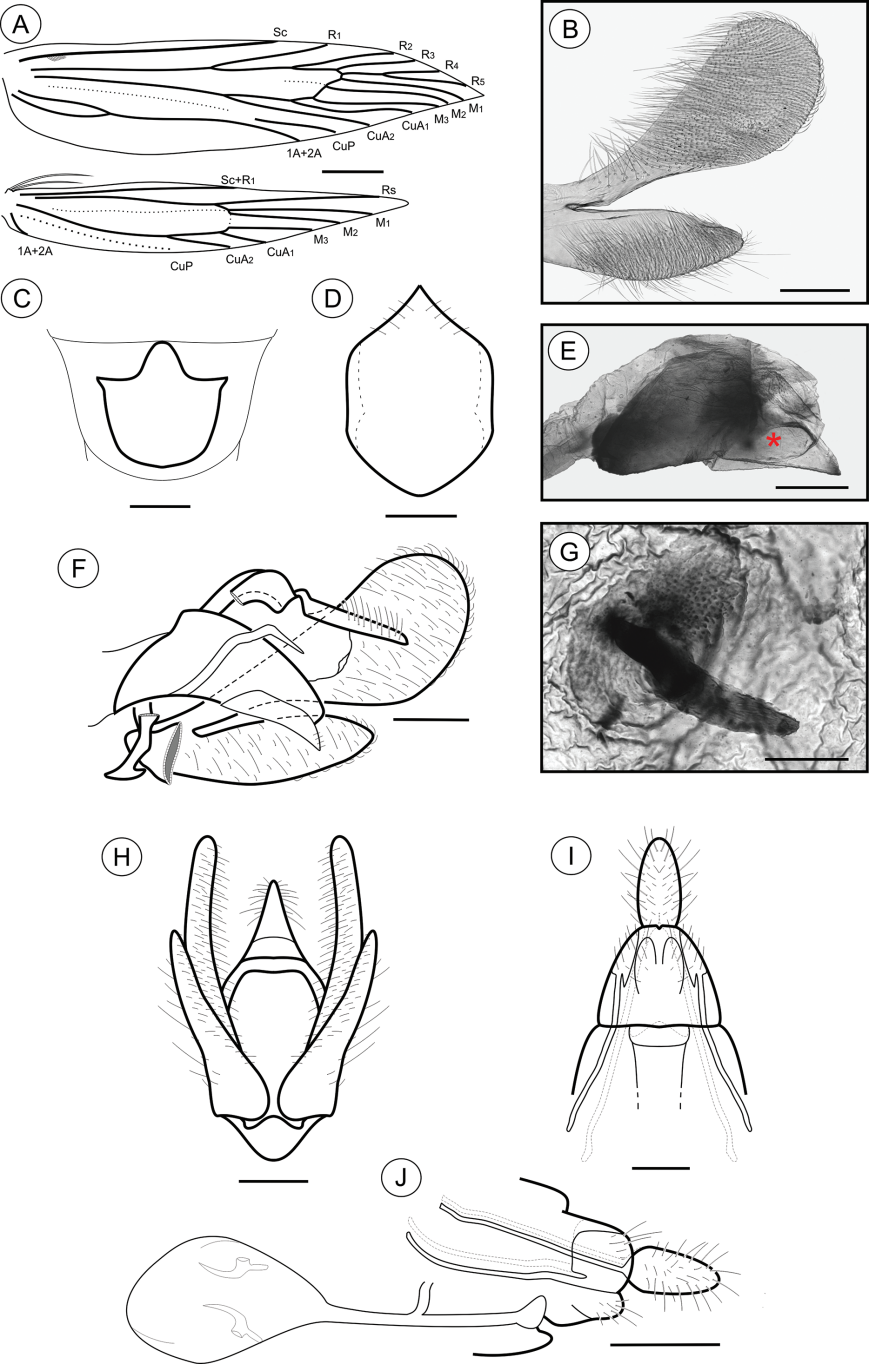
*Palaeomystella tavaresi* adult morphology: **A** wings **B** male valva, mesolateral view **C** male eighth sternum, ventral view **D** juxta, ventral; **E** aedeagus, lateral view (asterisk indicates attached juxta-lobes) **F** male genitalia, lateral view **G** signum, internal view **H** male genitalia, ventral view (transtilla, aedeagus and juxta not illustrated) **I** female genitalia, ventral view (corpus bursae not illustrated) **H** female genitalia, lateral view. Scale bars = 1 mm; 200, 250, 50, 200, 100, 200, 200 and 250 µm; 0.5 mm, respectively.

**Figure 9.**
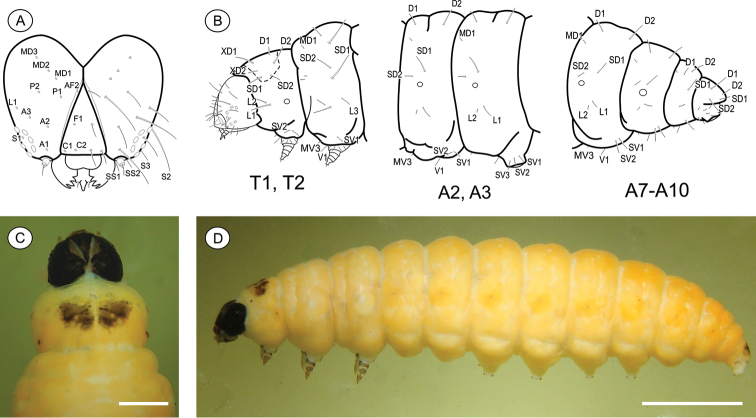
*Palaeomystella tavaresi* last larval instar: **A** cephalic chaetotaxy, frontal view **B** thoracic and abdominal chaetotaxy, lateral view **C** head and prothoracic shield in detail, dorsal view **D** body, lateral view. Scale bars = 50 µm, 1 mm, respectively.

**Figure 10.**
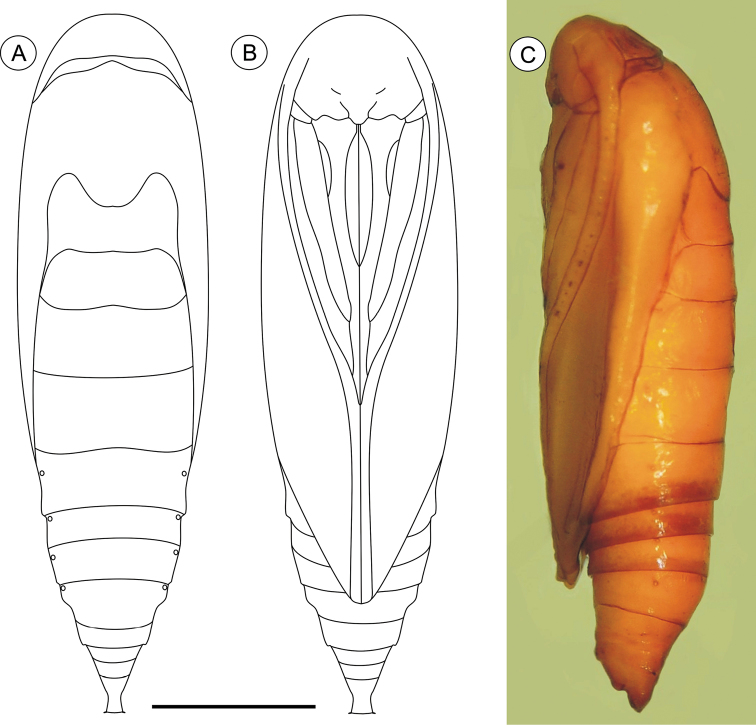
*Palaeomystella tavaresi* pupa, in dorsal (**A**), ventral (**B**) and lateral (**C**) views, respectively. Scale bar = 1 mm.

**Figure 11.**
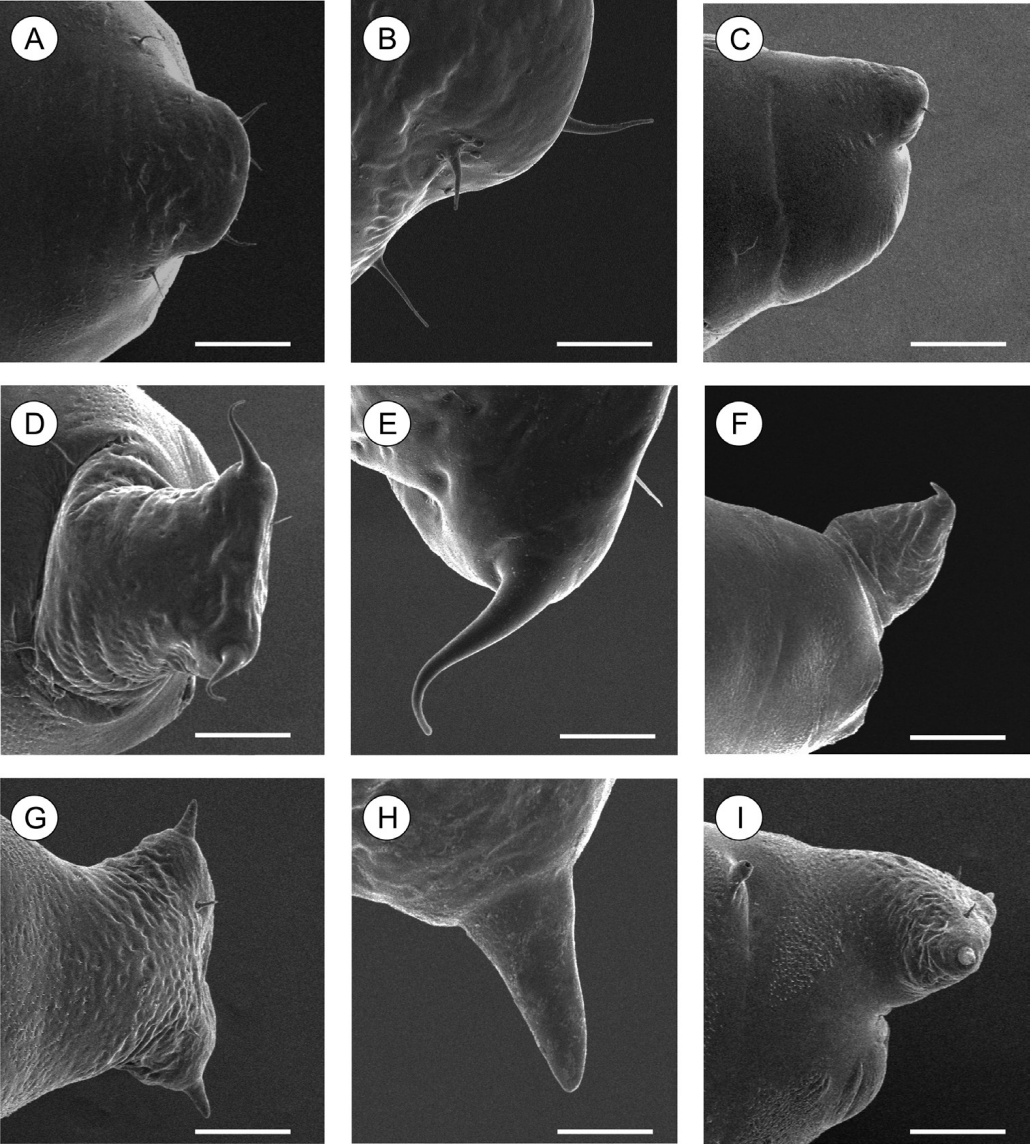
Scanning electron micrographs of *Palaeomystella* species pupal cremaster, in dorsal view (left column), apical process in detail (central column) and lateral view (right column): **A–B** *Palaeomystella fernandesi* **C–D** *Palaeomystella rosaemariae*; **E–F** *Palaeomystella tavaresi*. Scale bars = 100, 20, 200, 100, 20, 200, 100, 20, 200 µm, respectively.

**Figure 12.**
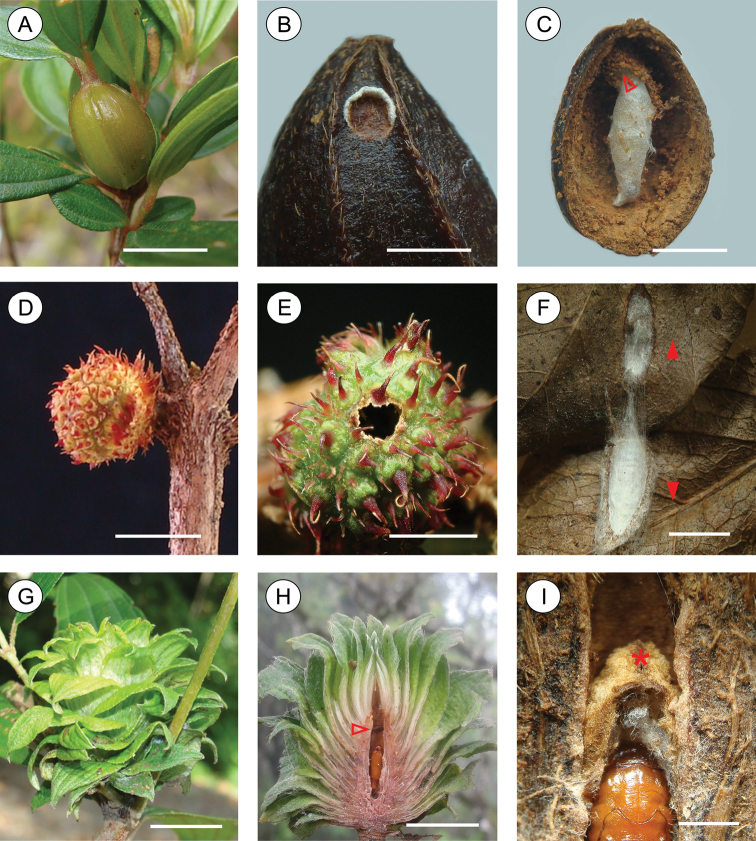
Galls induced by *Palaeomystella* species: **A–C** *Palaeomystella fernandesi* **D**–**F** *Palaeomystella rosaemariae* **G**–**I** *Palaeomystella tavaresi* **A** on *Tibouchina sellowiana*, general view **B** operculum made by last-instar larva on gall surface before pupation; **C** pupal cocoon in a dissected gall (arrow indicates the operculum shown in **B**) **D** on *Tibouchina asperior*, general view **E** exit hole made by last larval instar on gall surface **F** pupal cocoon constructed between two leaves, uncovered by pulling them apart (direction indicated by arrows) **G** on *Tibouchina fissinervia*, general view **H** longitudinally dissected gall, showing gall chamber (arrow indicates position of exit orifice on cocoon) **I** internal chamber in detail, showing the exit orifice on cocoon (asterisk). Scale bars = 10, 2, 4, 10, 5, 4, 10, 10, 2 mm, respectively.

Figs 1E–F , 8 – 10 , 11G–I , 12G–I

Walshia sp. Lima 1945: 303–305, figs 180, 183, 184, misidentification.

#### Diagnosis.

Closest to *Palaeomystella rosaemariae*, sharing with this species a pronounced palmate costa of the valve and a bladelike signa. As already mentioned, these characteristics differentiate them from all other species of *Palaeomystella* except *Palaeomystella oligophaga*. This species, however, has the forewings with R_4_-R_5_ fused and the hindwing with M_1_ and M_2_ stalked from the remnant chorda of the cell (Becker and Adamski 2008). *Palaeomystella tavaresi* differs from *Palaeomystella rosaemariae* by having: 1) adults with body covered with pale-brown scales tipped with brown, and brown scales; 2) males with latero-anterior margin of eighth sternum anteriorly expanded medially into a stout, rounded lobe; valva with distal portion of costa wider; juxta longer than wide, anteriorly convex; 3) females with signa having inward projections shorter, straight and stout; 4) pupa with cremaster slightly bifurcated and posteriorly directed, with a latero-apically located pair of blunt spines; 5) galls of rosette type, induced on apical/terminal buds of *Tibouchina fissinervia* shoots, causing growth of clustered short leaves with a cylindrical gall chamber.

#### Description.

**Adult** (Figs 1E–F). Sexes similar, forewing length 7.02 to 9.23 mm (n = 8). *Head*: Frons pale brown; vertex with pale-brown scales tipped with brown (Fig. 1F); labial palpus pale brown, basal segments angled laterally, terminal segment slightly angled upward; antennae brown; proboscis yellowish brown. *Thorax*: Tegula and mesonotum (Fig. 1F) with brown scales tipped with dark brown, posterior scales paler brown; fore and midlegs dark brown; hindlegs pale brown, tibia and tarsus with intermixed dark-brown scales. Forewings (Figs 1E, 8A): lanceolate, with 13 veins; L/W index ~ 4.4; dorsally covered with brown scales, intermixed with dark-brown scales tipped with black, and pale-brown scales; a narrow, ill-defined, dark-brown streak bisects the wing longitudinally from base to a brown, subapical, crescentic marking, edged distally with dark-gray scales; 3 raised scale tufts located posterior to cubitus, in anal area, in line with midcell, and near tornal area, respectively; fringes pale brown; ventral side most uniformly covered with dark-brown scales; discal cell closed, ~ 0.7× length of forewing; ending near 1/5 wing margin; Sc ending ca. 1/3 anterior margin; R 5-branched; R_1_ ending near 1/4 of wing margin; R_4_ and R_5_ stalked ca. 1/2 distance from cell apex; M 3-branched; CuA 2-branched; CuP weak proximally and not stalked, with 1A+2A that is well developed, extending more than half length of posterior margin. Hindwing (Figs 1E, 8A): strongly lanceolate, with 9 veins; L/W index ~ 5.4, ~ 0.84 forewing in length; scales light brown on both sides; fringes pale brown; frenulum a single acanthus on male, with two parallel-sided acanthi in female. Sc+R_1_ ending ca. 1/2 anterior margin; Rs ending near end of anterior margin; M 3-branched, with M_1_ and M_2_ stalked near Rs; CuA 2-branched; CuP weakly sclerotized, ending at 1/2 posterior margin; 1A+2A well developed, ending near basis of posterior margin. *Abdomen* (not illustrated): scales pale brown intermixed with gray scales, with transverse irregular rows of spiniform setae on terga 2–7 in both sexes. Eighth sternum (Fig. 8C) expanded anteromedially into a stout, rounded lobe, associated with a subtrapezoidal sternite.

**Male genitalia** (Figs 8B, D–F, H). Uncus narrow, separated from tegumen by a narrow membranous area, laterally setose (Figs 8F, H); tegumen narrow; vinculum widened ventrally; transtilla a short, flat fig; aedeagus tubiform, curved ventrally, short (2× longer than wide), slightly wider basally (Figs 8E–F); vesica bearing several stout cornuti; juxta (Fig. 8D) attached to distal portion of aedeagus (Fig. 8E), as long as wide, with convex basal margin and pointed distally; valva (Fig. 8B) covered by several long setae, divided near 1/3 from the basis, with sacculus spatulate, tapering distad, and costa long, palmate, gradually constricted basad.

**Female genitalia** (Figs 8G, I–J). Papillae anales connected dorsally, setose (Figs 8I–J); anterior apophyses similar in length to posterior, slightly curved apophyses; sterigma divided into a bandlike tergum and a distally bilobed sternum, deeply and narrowly emarginate medially; ostium bursae of small size, wider than long; ductus bursae membranous, longer than corpus bursae, with ductus seminalis inserted medially; corpus bursae an elongate sac, bearing two stout, straight, bladelike signa connected to crescentic figs located in the wall (Figs 8G, J).

#### Type material.

Holotype ♂: **Brazil:** Reserva Serra Bonita, 15°23'30"S, 39°33'57"W, 832 m, Camacan, BA, Brazil. Adults preserved dried and pinned, reared from galls induced on *Tibouchina fissinervia* (Schrank & Mart. ex DC.) Cogn. (Melastomataceae) by G.R.P. Moreira, 15–21.X.2013, LMCI 230-05, donated to DZUP (29.415). **Paratypes:** same data, 17–23.II.2013, LMCI 209; 1♂ (LMCI 209-31), 1♀ (LMCI 230-20), donated to DZUP (29.416 and 29.417, respectively); 1♂ (LMCI 230-06), 2♀ (LMCI 230-09 and 22) donated to VOB.

#### Other specimens examined.

Adults dried and pinned, collected in light traps at the type locality, deposited in VOB: 1♂ (VOB 144730), -.VIII.2009, by F.L. Santos; 1♂ (VOB 146783, with genitalia mounted on slide), -.IX.2010, by V.O. Becker. Additional specimens, with the same collection data as the type material, deposited in LMCI: adults dried and pinned, 6♂ (LMCI 230-07, 15, 16, 17 and 21; LMCI 230-08, with genitalia in glycerin GRPM 50-57) and 6♀ (LMCI 230-10, 11, 12, 18 and 19; LMCI 230-23, with genitalia in glycerin GRPM 50-58). Slide preparations, mounted in Canada balsam: adults, 1♂ (GRPM 50-54), 1♀ (GRPM 50-55); wings, 1♂ (GRPM 50-53); larvae, 2 last instars (GRPM 50-56). Immature stages, fixed in Kahle-Dietrich’s fluid and preserved in 70% EtOH: 5 last-instar larvae (LCMI 209-13 and 14, and 230-2); 6 pupae (LMCI 209-7, 11, 18, and 230-1); 12 dissected galls (LMCI 209-21 and 22, 230-3 and 4). In tissue collection, 6 larvae (LMCI 209-06) fixed and preserved in 100% EtOH, at -20°C.

#### Immature stages.

**Last larval instar** (Fig. 6), 7.28 to 11.7 mm (n = 4). Cecidogenous, endophyllous, semiprognathous and tissue-feeder. Body subcylindrical, creamy white, changing to light yellow before pupation, with setae well developed. *Head* (Figs 9A, C–D): uniform dark brown, with two conspicuous unpigmented, irregularly shaped areas along ecdysial line; smooth, with shallow ridges; labrum shallowly notched; frons higher than wide, extending ca. 3/4 epicranial notch; six stemmata arranged in C-shaped configuration. Chaetotaxy (Fig. 9A): A-group trisetose; L-group unisetose; P-group bisetose; MD trisetose; C-group bisetose; F-group unisetose; AF-group bisetose; S-group trisetose; SS-group trisetose. A1, A3, P1 and S2 about equal in length, longest setae on head; C1, C2, F1, A2, AF2, L1 intermediate in length; AF1 absent; MD1–3 very reduced and aligned with each other. Antenna two-segmented. Mandibles broad, with four teeth and one seta on the outer surface; labium broad, with two-segmented palpus, the distal segment minute; spinneret parallel-sided; maxilla prominent. *Thorax and Abdomen* (Figs 9B–D): Prothoracic shield and anal fig irregularly marked with dark brown. Thoracic legs light brown. Prolegs on A3–A6 and A10 of equal size; crochets in a circle, uniserial and uniordinal. Thorax chaetotaxy: T1 with D-group bisetose, both located on the dorsal shield, D1 shorter than D2; XD-group bisetose, setae similar in length and both located on the dorsal shield; SD bisetose, located laterally on the dorsal shield; L group bisetose, L1 longer than L2; SV-group bisetose, posteroventral to L2, SV1 slightly longer than SV2; V-group unisetose. T2 and T3 with D- and SD-groups bisetose, median-transversely aligned; D2 and SD1 similar in length, and longer than D1 and SD2 respectively; L trisetose, L3 posterior to L1 andL2, similar in length to L1; SV unisetose; V unisetose. Abdomen chaetotaxy: D-group bisetose; A1–9 with D2 slightly longer than D1, and A10 with D1 longer than D2; SD-group bisetose, A1–7 with SD1 slightly longer than SD2, A10 with SD2 longer than SD1, SD2 absent in A9; A1–8 with L-group bisetose, L1 longer than L2, L2 absent in A9; A1–8 with SV-group trisetose, SV3 absent in A7–9; V-group unisetose.

**Pupa** (Figs 10A–C, 11G–I), 6.37 to 8.84 mm (n = 5), elongate-oval in dorsal and ventral views, widest in the thoracic region. Integument light amber-colored, mostly smooth, with a few scattered microsetae dorsally. Frontoclypeal suture not evident. Labrum U-shaped, weakly defined. Labial palpi long; antennae arched anteriorly and separate, approximate and parallel posteriorly to distal margins of maxillae, surpassing apical margin of forewings; maxillae extending distally between sclerites of midlegs; femora of midleg not fused distally; femora of foreleg extending beyond widest part of labial palpi. Cremaster (Figs 11G–I) short, slightly bifurcated and posteriorly directed, bearing latero-apical pair of blunt spines.

#### Distribution.

*Palaeomystella tavaresi* is known only from the type locality, in preserved fragments of the Atlantic Rain Forest at the Serra Bonita Reserve, Camacan, Bahia, Brazil.

#### Host plant.

*Tibouchina fissinervia* (Schrank & Mart. ex DC.) Cogn.(Melastomataceae), a pioneer tree species that grows up to 20 m tall in the Atlantic Rain Forest, where it is endemic, ranging from Bahia to São Paulo (Freitas 2011). In the Serra Bonita Reserve, these trees are relatively common at higher altitudes, above 600 m, growing spontaneously in areas that were formerly cleared for agriculture and along trails and in clearings in pristine forests, resulting from the fall of other trees.

#### Life history

(Figs 12G–I). Gall prosoplasmatic histioid (Küster, in Meyer 1987), of the rosette type (internal length from 18 to 31 mm; n = 6), induced on growing shoots causing growth of clustered short leaves (Fig. 12G); green, progressively darkening during senescence, after emergence of the moth; unilocular, unilarval. A longitudinal, narrow, cylindrical chamber is formed in the middle (Fig. 12H), where the larva develops and pupate. The mature last instar larva constructed a brown silk plug/gate near the middle of the chamber consisting of two convex hatches that open horizontally (Fig. 12I). Then it constructed a flimsy silk cocoon in the proximal sector of the chamber, where pupation occurred. To exit the cocoon the adult pushed the hatches open, and emerged through the terminal leaflets of the gall. At the type locality, the galls occur in small numbers on *Tibouchina fissinervia* trees, occasionally in groups of a few per plant. Under laboratory conditions, mature galls collected in the spring (October) had the adults emerging ca. 15 days later.

#### Etymology.

*Palaeomystella tavaresi* is named in memory of the Jesuit priest Joaquim da Silva Tavares, a Portuguese naturalist and a pioneer in the study and description of Brazilian cecidology (Tavares 1917).

#### Remarks.

Lima (1945: 304, 305, Figs 180, 183, 184) illustrated the gall, wing venation and male genitalia of a species that he identified as a member of *Walshia* Clemens (Cosmopterigidae), reared from galls on branches of a species of *Tibouchina* collected in Petrópolis, Rio de Janeiro. The gall, genitalia and wing venation appear almost identical to those of *Palaeomystella tavaresi* and very likely represent the same species. Tavares (1917: 31, pl. 1, Figs 1, 2) described a stem gall, also from a *Tibouchina* sp., collected at Tijuca and Petrópolis, Rio de Janeiro, which exactly resembles the galls of *Palaeomystella tavaresi*. However, his description of the moths as “shiny, brunneous, with several golden spots on the upper side of forewings” does not match the one described here.

#### Molecular phylogeny.

**Figure 13.**
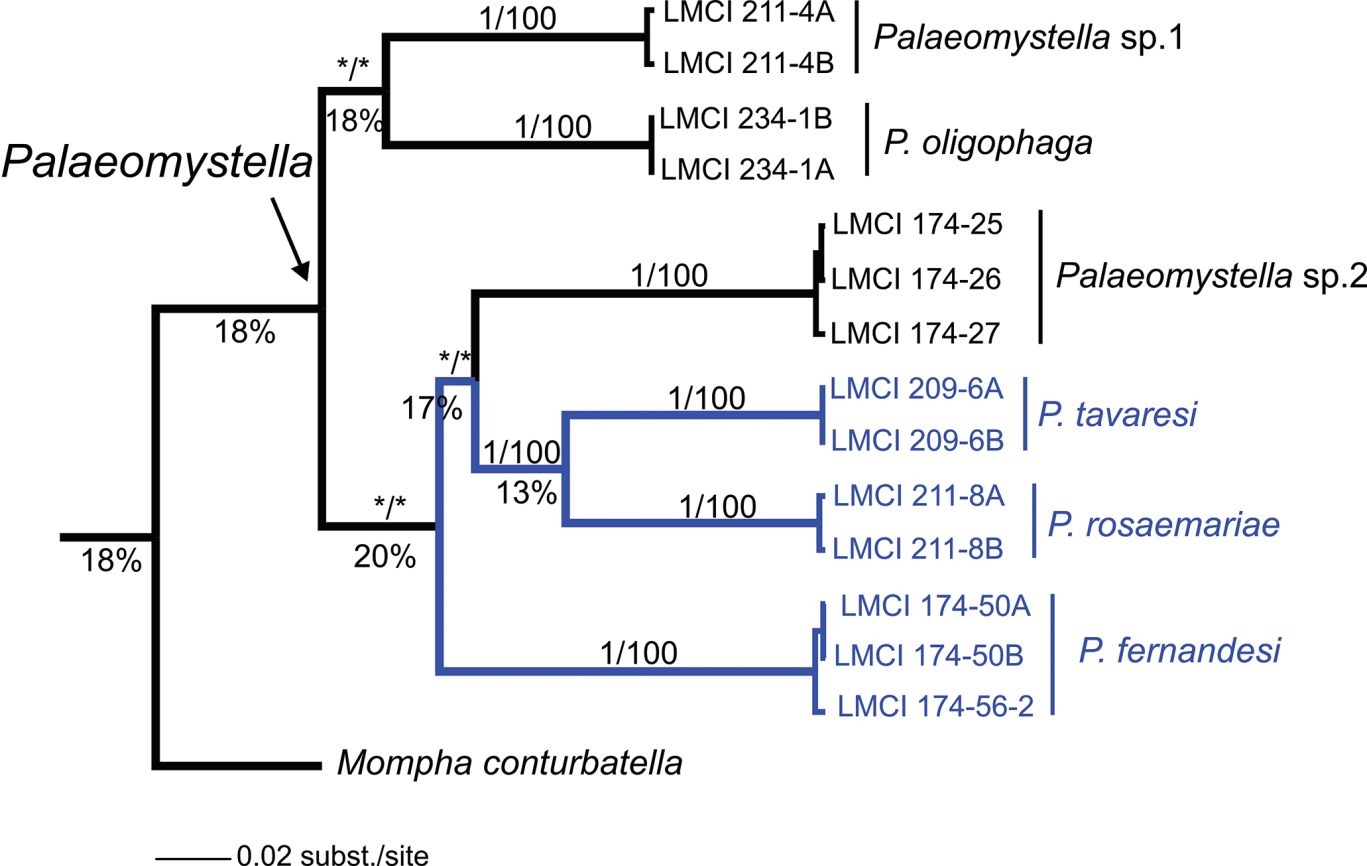
Bayesian inference tree for *Palaeomystella*, based on 660 bp of the mitochondrial cytochrome oxidase *c* subunit I gene (CO-I). Numbers above branches indicate support values > 0.8/60 for Bayesian Posterior Probability (BPP)/Bootstrap - for Maximum Likelihood (ML) (see Material and methods); those located below represent the percentage of evolutionary divergence between clades. Asterisk indicates support < 0.80/60 for BPP and ML, respectively.

A total of 660 nucleotide sites were analyzed for species of *Palaeomystella* from different host plants, and 211 (32%) of these were variable. According to the phylogenetic hypothesis proposed here, all species were recovered as monophyletic lineages within the *Palaeomystella* group of Momphidae, in both methods of inference (BI and ML) with full branch support (Fig. 13). Regarding internal relationships, *Palaeomystella rosaemariae* was placed as a sister of *Palaeomystella tavaresi* with strong posterior probability (= 1) and bootstrap (=100). *Palaeomystella fernandesi* was more distantly related, although with low branch support (< 0.8, posterior probability; < 70, bootstrap). Despite the strong internal statistical branch support of the three new lineages of momphids, the external relationships for *Palaeomystella* were poorly resolved (i.e., position of clades), and even the monophyly of the genus lacks statistical support. *Mompha* was used to root the tree, but its position as a sister clade of *Palaeomystella* was not supported (Fig. 13). The evolutionary divergence observed between comparisons of pairs of species was markedly high, showing greater genetic variation in this group of momphids (Table 2), particularly between clades (Fig. 13). An average of 18% (± 3%) of K2P differences was found between species of *Palaeomystella*, ranging from 14 (± 2%) to 24% (± 3%). The maximum divergence observed among clades was 20%, found between *Palaeomystella fernandesi* and the clade formed by *Palaeomystella rosaemariae* + *Palaeomystella tavaresi* + *Palaeomystella* sp. 1 (Fig. 13). The genetic divergence within *Palaeomystella* (ca. 18%) was greater than between this genus and *Mompha* (16%).

**Table 2.** Estimates of evolutionary divergence between sequences based on 660 base pairs of the cytochrome oxidase *c* subunit I (CO-I) gene using the Kimura 2-parameter model. Mean number (± standard error) of base substitutions per site over all sequence pairs between groups, obtained by a bootstrap procedure of 1000 replicates is shown. The analysis involved the three new *Palaeomystella* species described in this study (marked in bold), two undescribed taxa (sp. 1 and sp. 2), one currently recognized taxa (*Palaeomystella oligophaga*) and the outgroup (*Mompha*).

	1.	2.	3.	4.	5.	6.
1. *Mompha conturbatella*						
2. *Palaeomystella* sp. 1	0.18±0.02					
3. *Palaeomystella* sp. 2	0.20±0.02	0.23±0.03				
**4. *Palaeomystella fernandesi***	**0.21±0.02**	**0.23±0.02**	**0.24±0.03**			
**5. *Palaeomystella tavaresi***	**0.18±0.02**	**0.22±0.03**	**0.20±0.03**	**0.22±0.03**		
**6. *Palaeomystella rosaemariae***	**0.16±0.02**	**0.24±0.03**	**0.20±0.03**	**0.21±0.03**	**0.14±0.02**	
7. *Palaeomystella oligophaga*	0.14±0.02	0.18±0.02	0.25±0.03	0.17±0.02	0.19±0.02	0.16±0.02

## Discussion

As pointed out by Becker and Adamski (2008), for the other congeneric members it is almost certain that species described herein belong to the Momphidae lineage. They are, however, tentatively placed within *Palaeomystella*, based upon shared characters of the genitalia and on the monophyly found in the preliminary molecular analysis carried out in this study. They differ from the type-species *Palaeomystella chalcopeda*, since in all cases the female genitalia have anterior and posterior apophyses that are similar in length. As illustrated by Becker (1999), in *Palaeomystella chalcopeda* the posterior apophyses are almost twice as long as the anterior apophyses. Thus, the male genitalia, immature stages, and galls, if any, of the type species of *Palaeomystella* remain unknown, which prevents any decision about its taxonomic status.

The genetic variability found herein at the generic level (maximum distance among *Palaeomystella* clades = 20%; average among species = 18%), by using a few putative species, strongly suggests that there are several gaps in diversity in the analysis. This pattern results in part from low collection efforts and the small number of taxonomic studies on this lineage in the area. Alternatively, the result could be associated with the single marker used. Although loci used in DNA barcoding are not the most suitable for making broad inferences on phylogenetic relationships (Müller et al. 2013), we can still reveal a scenario of monophyletic status and diversity at the generic level using this molecular evidence (see Lees et al. 2013). The use of more gene loci could be an alternative to shed light on the phylogenetic relationships at the species level and on the status of *Palaeomystella* at the generic level. In other families of microlepidopterans, more loci have been used to solve phylogenetic problems at different taxonomic levels, for example in Gracillariidae (Kawahara et al. 2011) and Gelechioidea (Heikkilä et al. 2013). This effort might still not be entirely adequate in the case treated here, since a greater, unknown diversity apparently exists, as can be observed in the significantly different lineages found. In other words, it is suggested first to direct efforts toward field sampling, and then to sample at least a few more loci in order to better understand the relationships within this group of momphids.

As mentioned above, the diversity of moth-induced melastome galls is in fact much greater that presented here (e.g. Hanson et al. 2014). Several species belonging to this group, in addition to the two that we included in the molecular analyses, are present in our collections and still await description. Furthermore, in contrast to our expectations the species described here are not only similar from a gross morphology perspective, but share several fine-scale morphological characteristics with those belonging to *Mompha* Hübner. For example, the divided male valva into two sections and the female bursa with a bladelike signa are also found within the latter genus (e.g., Hodges 1998, Wagner et al. 2004). A pupal cremaster bearing spines, similar to those described in this study, is also found among the Palearctic species of *Mompha* (e.g., Patočka and Turčani 2005). Contrary to what is known for the larvae of two species of *Palaeomystella* that were proposed by Adamski and Becker (2008), all species described here are bisetose regarding the prothoracic L-group setae, a characteristic of *Mompha* species (Stehr 1987, Wagner et al. 2004). Also in contrast to their findings, the present species do not show a reduction in the number of setae on the anal fig. Thus, the generic status of the species described in this study may change in the future, pending descriptions of additional taxa, further studies of phylogeny, and taxonomic revision of the family. A decision concerning this matter would be precipitate at present, as *Mompha* is similarly a poorly known genus, with species that are difficult to collect and have a wide variation of feeding habits (including cecidogeny). Therefore, several species either remain undescribed or lack taxonomic descriptions that are sufficiently detailed to allow comparisons. Furthermore, DNA sequences for only a few species are available, and as with *Palaeomystella*, show a wide range of evolutionary divergence (4–14%) (for discussion, see Emery et al. 2009).

This study illustrates further variation in gall morphotypes. Such variation has long been known to exist among Melastomataceae galls (Tavares 1917, Houard 1933), confirming that they are associated with different species of lepidopteran inducers. At least two of the galls described here may have appeared before in the literature, but none of them has been accurately associated with any cecidogenous species. The fusiform type induced by *Palaeomystella fernandesi* on *Tibouchina sellowiana* was reported by Toma and Mendonça (2013), in a gall survey that they carried out at the type locality, as being induced by an unidentified member of Gelechioidea. As already mentioned, depending upon the time of year, most such galls are associated in the field with a specialized kleptoparasitic gelechiid moth, with which the cecidogenous species may be confounded (Luz et al. 2014). Galls similar to the rosette type induced by *Palaeomystella tavaresi* in *Tibouchina fissinervia* were illustrated by Tavares (1917) and Houard (1933) for *Tibouchina* sp. in Rio de Janeiro State. Lima (1945) showed the same type of gall for an unidentified species of *Tibouchina*, also in Rio de Janeiro, but as mentioned above, he erroneously associated it with an unidentified species of *Walshia* (Cosmopterigidae). Furthermore, galls of the same rosette type as those of *Palaeomystella tavaresi*, apparently induced by unidentified species of Lepidoptera, were shown by Maia and Fernandes (2004) on *Miconia theizans* (Bonpl.) Congn. occurring in Minas Gerais State, and by Santos et al. (2011) and Isaias et al. (2013) on *Henriettea succosa* (Aubl.) DC. in Pernambuco State. Further studies should be conducted to determine whether such galls are induced by *Palaeomystella tavaresi* or by closely related species.

The present results also demonstrate the existence of considerable variation in life-history styles for the pupal stage of *Palaeomystella* species, which should be taken into account in future studies. That is, last-instar larvae may remain endophylous until pupation in either sessile (*Palaeomystella tavaresi*) or dehiscent (*Palaeomystella fernandesi*) galls, or may leave them to pupate in leaf litter (*Palaeomystella rosaemariae*). Although varying little in the general integumentary morphology, their pupae show considerable variation in the size and shape of the cremaster which may provide useful characters for future species identifications. Unfortunately, the other known species of *Palaeomystella* (Becker and Adamski 2008) were not described under the scanning electron microscope, and thus cannot be closely compared. These structures are supposedly used to anchor the pupa laterally to the cocoon/pupal chamber. They have likely evolved in conjunction with the habit of emerging of the adults in the pupation sites, which apparently first appeared in the Lepidoptera evolution within the Gelechioidea (e.g. Powell 1973, Becker 1982). Variation in chaetotaxy among species may also prove to be useful to identify different lineages within *Palaeomystella*. For example, in addition to the variation in number of setae described by Adamski and Becker (2008) for the prothoracic L-group and anal fig, a numerical variation in chaetotaxy was also detected on the head (AF1 seta), thorax and abdomen (SD setae).

## Supplementary Material

XML Treatment for Palaeomystella fernandesi

XML Treatment for Palaeomystella rosaemariae

XML Treatment for Palaeomystella tavaresi
